# 1-stearoyl-2-arachidonoyl-driven B Cell metabolic dysregulation in chronic rhinosinusitis with nasal polyps: insights from Mendelian randomization and single-cell RNA sequencing

**DOI:** 10.3389/fphar.2025.1719897

**Published:** 2025-10-30

**Authors:** Jian Wu, Yifang Sun, Shuyue Wang, Qian Zhang, Ying Lin, Caipeng Liu, Maomao Ai, Feng Yu, Lei Cao

**Affiliations:** ^1^ Department of Otorhinolaryngology-Head and Neck Surgery, Guangzhou Red Cross Hospital of Jinan University, Guangzhou, Guangdong, China; ^2^ Department of Ophthalmology, Guangzhou Red Cross Hospital of Jinan University, Guangzhou, Guangdong, China; ^3^ Department of Otorhinolaryngology, The Forth People’s Hospital of Guiyang, Guiyang, Guizhou, China

**Keywords:** CRSwNP, B-cell, metabolism, immunity, Mendelian randomization, single-cell RNA sequencing

## Abstract

**Background:**

Chronic rhinosinusitis with nasal polyps (CRSwNP), an inflammatory condition of unclear etiology, may involve immune dysregulation and metabolic alterations.

**Methods:**

Utilizing Mendelian randomization, we investigated causal links between CRSwNP and profiles of 731 immune cell types and 1,400 metabolites. Single-cell RNA sequencing (scRNA-seq) was employed for cell type identification and transcription factor analysis. Metabolic profiling characterized cellular subpopulations, while Gene Set Enrichment Analysis (GSEA) and machine learning pinpointed key genes functionally linked to immune and inflammatory pathways (categorized via WGCNA and Metascape).

**Results:**

We identified expression of HLA-DR on CD33− HLA-DR + B cells and the lipid metabolite 1-stearoyl-2-arachidonoyl as risk factors for CRSwNP. scRNA-seq further revealed these specific B cell subpopulations exhibit metabolic levels linked to immune responses. Bulk RNA analysis confirmed upregulation of genes CD27 and DERL3, while machine learning identified a signature of ten key genes showing positive correlation with B cell regulatory functions.

**Conclusion:**

This integrated study advances understanding of immune-metabolic crosstalk in CRSwNP pathogenesis, highlighting the role of metabolite-influenced B cell subsets in shaping the immune microenvironment, thereby suggesting novel therapeutic targets.

## 1 Introduction

CRSwNP is a persistent inflammatory disorder affecting the nasal mucosa and paranasal sinuses. Characterized by infiltration of diverse inflammatory cells, this condition typically presents with symptoms including persistent nasal congestion, rhinorrhea, pain, and hyposmia/anosmia (Bachert et al., 2021). Studies have shown that the prevalence of CRSwNP is about 1.1% in the United States and 2.1%–4.4% in Europe (Laidlaw et al., 2021). Notably, CRSwNP affects about 20%–30% of patients with chronic rhinosinusitis (CRS), which imposing a substantial socioeconomic burden and significantly impairing patients’ quality of life. Current treatment for CRSwNP mainly includes corticosteroid drugs and endoscopic sinus surgery; however, these approaches are associated with potential adverse effects (e.g., from corticosteroids) and notably high recurrence rates following surgery (Hox et al., 2020).

While the precise etiology of CRSwNP remains incompletely elucidated, the roles of immune dysregulation and metabolic perturbations in its pathogenesis have garnered increasing attention (Veloso-Teles et al., 2019). Emerging evidence suggests metabolites can modulate immune cell function (Veloso-Teles et al., 2019). CRSwNP is commonly stratified into eosinophilic (ECRSwNP) and non-eosinophilic (non-ECRSwNP) subtypes based on tissue eosinophilia levels (Cao et al., 2009). Notably, linoleic acid has been implicated in eCRSwNP pathogenesis through its suppressive effects on eosinophilic inflammation (Ma et al., 2021). Furthermore, M2 macrophages contribute to CRSwNP development via complex immune responses and tissue remodeling processes, with several M2-associated hub genes identified as critical contributors (Zhu et al., 2022). Interestingly, resolvin D1 (RvD1), a specialized pro-resolving mediator, promotes macrophage polarization towards this M2 phenotype (Vickery et al., 2021). B-cell responses, including proliferation, antibody production, and aberrant pathway activation, are increasingly recognized as key drivers in CRSwNP (Bai and Tan, 2023), and heightened expression of B-cell activating factor (BAFF) is strongly linked to postoperative recurrence (Zhang et al., 2022). Intriguingly, RvD1 and its precursor 17-HDHA have been shown to influence naïve B-cell differentiation (Kim et al., 2015). Collectively, these findings underscore the intricate interplay between immunity and metabolism in CRSwNP. This relationship is further highlighted by distinct metabolic profiles observed across CRSwNP subtypes. Specific metabolites, including maresins, specialized pro-resolving lipid mediators (SPMs), and linoleic acid, are considered potential diagnostic biomarkers (Guo et al., 2023), while enzymes and metabolites involved in fatty acid metabolism represent promising therapeutic targets (Miyata et al., 2019). Nevertheless, a comprehensive understanding of the specific metabolic-immunoregulatory mechanisms governing CRSwNP pathogenesis is still lacking.

In this study, we employed an integrated approach utilizing Mendelian randomization, single-cell RNA sequencing (scRNA-seq), and transcriptome analysis to elucidate the underlying immunometabolic regulatory mechanisms in CRSwNP. Specifically, Mendelian randomization was applied to identify metabolites and immune cells exhibiting causal relationships with CRSwNP. Subsequently, integrated scRNA-seq and transcriptomic analyses were leveraged to delineate the specific immunomodulatory roles of key metabolites within the CRSwNP context.

## 2 Methods

### 2.1 Mendelian randomization analysis

Mendelian randomization (MR) utilizes genetic variants as instrumental variables, adhering to the principle of Mendelian inheritance, to infer causal relationships between exposures and outcomes while mitigating confounding biases. In this study, we employed a two-sample MR design to investigate the causal effects of plasma metabolites and circulating immune cells on CRSwNP (Zhu et al., 2018). The results of which Mendelian randomization satisfy three core assumptions: (1) Association assumption: The instrumental variables (IVs) must be strongly associated with the exposure. (2) Independence assumption: The IVs must be independent of confounding factors that influence the exposure-outcome relationship. (3) Exclusion restriction assumption: The IVs must influence the outcome solely through the exposure pathway. Primary MR analyses were conducted using the inverse variance weighted (IVW) method implemented in the TwoSampleMR package. We defined plasma metabolites and peripheral blood immune cell phenotypes as exposures, and CRSwNP as the outcome. Exposure data for 1,400 plasma metabolites were sourced from the genome-wide association study (GWAS) by Chen et al. (2023). Exposure data for 731 circulating immune cell phenotypes, encompassing diverse developmental stages and cell types, were obtained from a comprehensive GWAS of peripheral blood immune cells (Orru et al., 2020). This dataset included absolute cell counts, relative cell counts, median fluorescence intensity (MFI) reflecting surface antigen expression, and morphological parameters. We performed MR analyses to identify metabolites and immune cell traits causally associated with CRSwNP risk. All reported associations underwent false discovery rate (FDR) correction for multiple testing. Ethical approval and participant informed consent were secured in the original studies providing the GWAS summary statistics used in this MR analysis. These data are publicly accessible via the GWAS catalog (https://www.ebi.ac.uk/gwas/) under the accession codes provided in the respective publications (Burgess et al., 2013).

### 2.2 Metabolomic profiling of CRSwNP

Nasal secretion samples were collected from 12 healthy individuals and 19 patients with CRSwNP. These samples were prospectively collected specifically for this study at Guangzhou Red Cross Hospital. This study was approved by the Medical Ethics Committee of Guangzhou Red Cross Hospital (Approval No: 2024-128-01), conducted in accordance with the Declaration of Helsinki, and all participants provided written informed consent. Clinical trial number: not applicable. Following nasal mucosa preparation using ephedrine and 2% tetracaine, sterile swabs were employed to gather secretions from the middle meatus, and these samples were subsequently stored at −80 °C. For metabolite extraction, 100 µL of each sample was combined with 700 µL of extraction solvent (methanol:acetonitrile:water = 4:2:1, v/v/v), vortexed, incubated at −20 °C for 2 h, and centrifuged at 25,000 × g for 15 min at 4 °C; the resulting supernatant was then evaporated and reconstituted in 180 µL of methanol:water (1:1, v/v). Liquid chromatography-mass spectrometry (LC-MS) analysis was conducted using a Thermo Q Exactive system equipped with a BEH C18 column, utilizing specific mobile phases for positive and negative ion modes and electrospray ionization (ESI) in full scan mode (m/z 70–1,050). Metabolites were identified by referencing the BGI HR-PMDB and mzCloud databases, and differential metabolites between the CRSwNP and normal groups were determined through principal component analysis (PCA), partial least squares discriminant analysis (PLSDA), and orthogonal partial least squares discriminant analysis (OPLSDA). Finally, pathway enrichment analysis was performed using the Kyoto Encyclopedia of Genes and Genomes (KEGG) database.

### 2.3 Single-cell RNA sequencing data processing

Guided by Mendelian randomization findings identifying metabolites and immune cells potentially causally linked to CRSwNP, we performed integrated analyses using scRNA-seq data. Single-cell transcriptomic data from CRSwNP patient samples were retrieved from the Gene Expression Omnibus (GEO) database under accession number GSE196169 (Bangert et al., 2022). Raw scRNA-seq data processing, quality control, normalization, dimensionality reduction, clustering, and annotation were conducted using the Seurat package (v4.3.0) (Zheng et al., 2017). Quality control filtering was applied as follows: cells expressing fewer than 200 genes or more than 4,000 genes were excluded, and cells with mitochondrial gene content exceeding 10% were also removed. Normalization and feature selection: Filtered gene expression counts were normalized using the NormalizeData function. Subsequently, highly variable genes (HVGs) were identified using FindVariableFeatures. Scaling, dimensionality reduction, and integration: Expression data were scaled (ScaleData) and centered prior to principal component analysis (PCA). To mitigate technical batch effects, batch correction was performed using the RunHarmony function (Stuart et al., 2019). The resulting integrated data were then subjected to uniform manifold approximation and projection (UMAP) for non-linear dimensionality reduction and visualization. Cell clustering and annotation: Cells were clustered using the shared nearest neighbor (SNN) modularity optimization approach (FindNeighbors followed by FindClusters) based on a selected number of principal components. Cell types were annotated using the SingleR package (Kanehisa et al., 2023) with reference datasets. Metabolic pathway scoring and differential expression analysis: Metabolic pathway activity was inferred and scored per cell using gene signatures derived from KEGG pathways. Finally, the FindMarkers function was employed to identify differentially expressed genes (DEGs) between biologically relevant subpopulations.

### 2.4 Bulk RNA-seq data processing

To validate immunometabolic regulatory genes identified through scRNA-seq analysis in CRSwNP, we performed integrative transcriptomic analysis using bulk RNA-seq datasets. Datasets GSE36830 and GSE23552 were retrieved from the GEO database (Stevens et al., 2015; Plager et al., 2010). The results after batch removal were visualized by the principal component analysis method to finally obtain a comprehensive data expression matrix, from which differential genes obtained from a single-cell analysis were extracted for subsequent analysis and modeling. GSVA was used for enrichment analysis, limma package was used for differential analysis, volcano plots obtained significant differentially expressed genes, and correlation analysis was used to explore the correlation changes of genes (Ritchie et al., 2015).

### 2.5 Gene set enrichment analyses

GSEA analysis assesses the distribution trend of genes in gene expression for a predefined gene set (Powers et al., 2018). GOBP_B_CELL_RECEPTOR_SIGNALING_PATHWAY was selected as a predefined gene set to evaluate the expression of B-cell receptor signaling pathways in differential genes and significant signaling pathways were selected by FDR q-value<0.25. Ssgsea allows for assessment of the enrichment of a gene set in a single sample (Jin et al., 2021). Immune infiltration analysis was performed by ssgsea. And the expression of genes in B cells was analyzed.

### 2.6 Single-cell transcription factor and cell communication analysis

Transcription Factor (TF) Analysis: B cell subsets isolated from scRNA-seq data were subjected to TF activity inference using the DoRothEA regulon database (Xu et al., 2024) via the run_viper function. Data scaling ensured comparability across cells. TF activity scores were computed using GetAssayData, with the top 20 most variable TFs visualized in heatmaps generated by the pheatmap package (minsize parameter = 4). Cell-Cell Communication Analysis: Intercellular signaling was investigated using CellCall (Zhang et al., 2021). Cellular populations were categorized as B cells and non-B cells. The analysis workflow included: (1) Object creation with CreateNichConObject; (2) Pathway-centric communication network identification via TransCommuProfile; (3) Statistical filtering (padj <0.05) and correlation analysis of significant ligand-receptor pairs.

### 2.7 Machine learning

We applied machine learning for feature selection and predictive modeling using the randomForest package (Ishwaran and Kogalur, 2010). Models were trained on the integrated bulk RNA-seq data from GSE36830 and GSE23552, with internal validation via 5-fold cross-validation and bootstrap resampling. This robust method efficiently handles high-dimensional data while maintaining noise resistance. Key genes were identified via feature importance ranking, followed by multivariable logistic regression modeling (glm function). Models underwent bootstrap validation (1,000 iterations) with performance quantification via sensitivity/specificity and receiver operating characteristic (ROC) curves (roc function), including AUC calculations. Decision curve analysis (decision_curve function) evaluated clinical utility, while nomogram visualization utilized the regplot package.

### 2.8 Immune infiltration analysis

Immune microenvironment profiling was conducted via the IOBR package (Newman et al., 2015), employing CIBERSORT (deconvo_cibersort) for relative immune cell quantification and MCP-counter for absolute cell abundance assessment. Analysis was restricted to DEGs meeting thresholds (adj. p < 0.05, |log2FC| > 0.5). Spearman correlations (psych:corr.test) with FDR correction analyzed gene-infiltration relationships, with identical methodology applied to inflammatory factors.

### 2.9 Clustering and Co-expression analysis

Consensus clustering (ConsensusClusterPlus) (Wilkerson and Hayes, 2010) defined molecular subtypes using k-means clustering (kmax = 10, 100 iterations, 80% sample retention). Cluster stability was evaluated via consensus matrices and item-consistency indices (calcICL). Weighted gene co-expression network analysis (WGCNA) (Langfelder and Horvath, 2008) identified co-regulated modules through: 1) Selection of top 2000 variant genes (goodSamplesGenes QC), 2) Sample outlier removal (hclust + cutreeStatic), 3) Soft-threshold determination (pickSoftThreshold), 4) Network construction (blockwiseModules), 5) Module eigengene extraction (moduleEigengenes), and 6) Module-trait correlation (cor + corPvalueStudent). Significant modules (p < 0.05) underwent functional annotation in Metascape (Zhou et al., 2019).

### 2.10 Statistical analysis

All analyses used R v4.2.3. Statistical methods included: Spearman correlation with FDR correction (psych:corr.test), data scaling (scaleData), recursive feature elimination with 5-fold cross-validation (rfe), group comparisons via Wilcoxon test, odds ratio calculation with forest plotting (forestploter), and Benjamini-Hochberg FDR adjustment. Significance was defined as two-sided p < 0.05 unless specified.

## 3 Results

### 3.1 Mendelian randomization analysis of immune cells and metabolites

**FIGURE 1:**
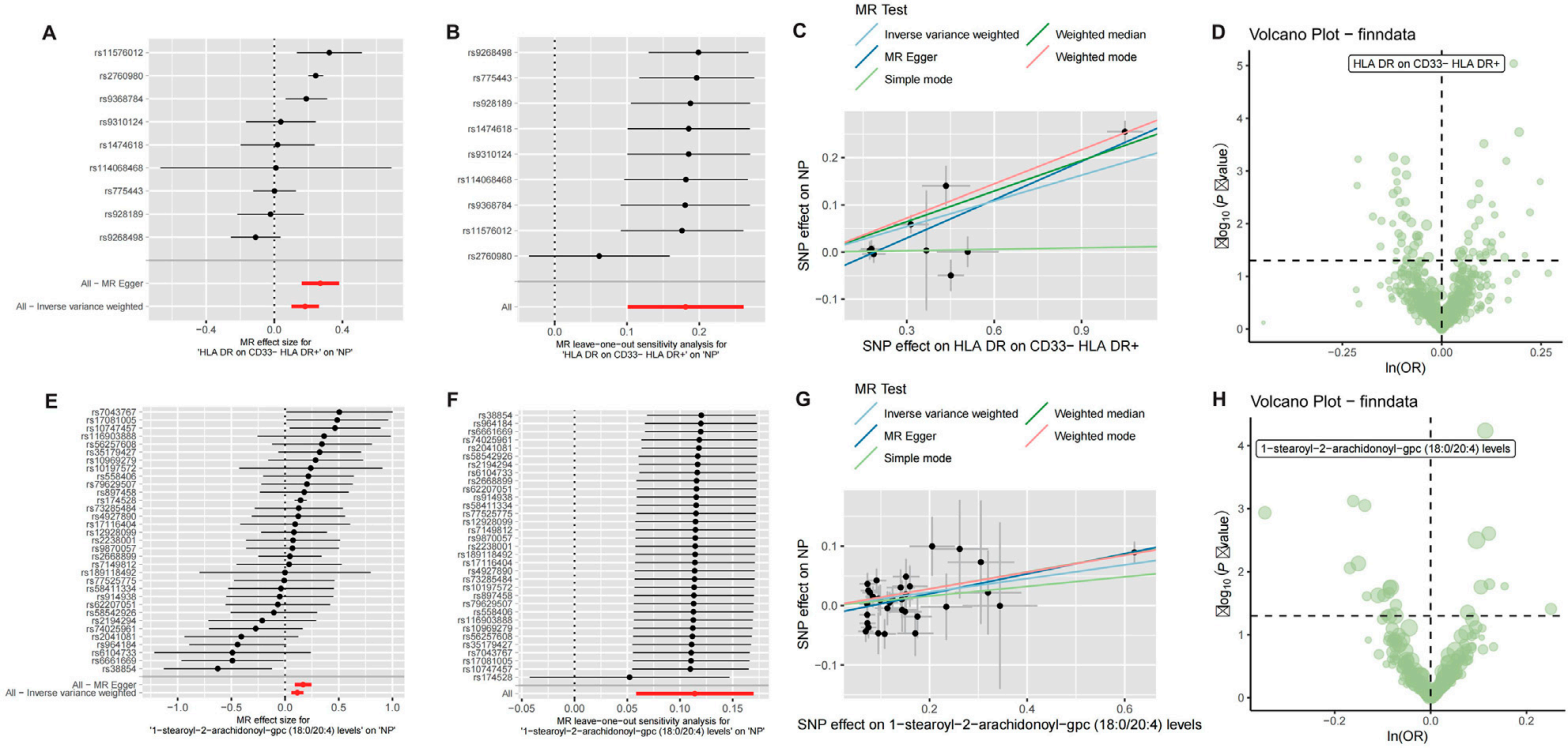
Mendelian randomization analysis of CRSwNP with immune cells and metabolites. **(A)** Forest plot of Mendelian randomization results for CRSwNP and HLA DR on CD33− HLA DR+. **(B)** Leave-out analysis for CRSwNP and HLA DR on CD33− HLA DR+. **(C)** Scatter plot of Mendelian randomization for CRSwNP and HLA DR on CD33− HLA DR+. **(D)** Volcano plot of Mendelian randomization for CRSwNP and immune cells. **(E)** Forest plot of Mendelian randomization results for CRSwNP and 1-stearoyl-2-arachidonoyl-gpc. **(F)** Leave-out analysis for CRSwNP and 1-stearoyl-2-arachidonoyl-gpc. **(G)** Scatter plot of Mendelian randomization for CRSwNP and 1-stearoyl-2-arachidonoyl-gpc. **(H)** Volcano plot of Mendelian randomization results for CRSwNP and metabolites.

Mendelian randomization analysis was performed to assess causal relationships between CRSwNP and 731 immune cell phenotypes or 1,400 plasma metabolites. Analysis of immune cells revealed that HLA-DR expression on CD33^−^ HLA-DR^+^ B cells exhibited a potential causal association with CRSwNP risk (beta = 0.181, FDR = 0.007; Figures 1A–D). For metabolites, 1-stearoyl-2-arachidonoyl showed evidence of significant causal effects on CRSwNP development (beta = 0.114, FDR = 0.015; Figures 1E–H). Complete MR results are documented in Supplementary Table S1, S2.

### 3.2 Integrated metabolomic profiling of CRSwNP

**FIGURE 2:**
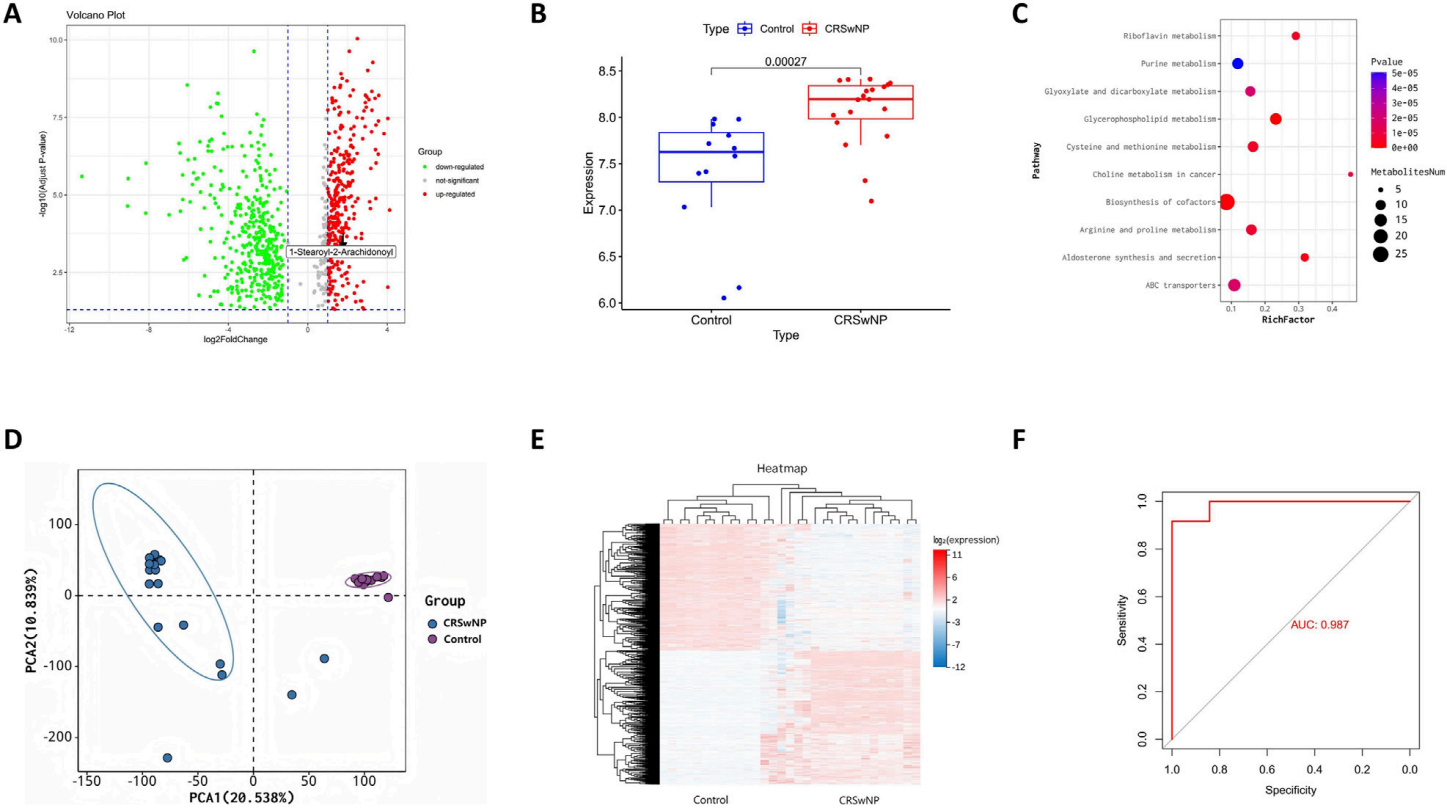
Integrated Metabolomic Profiling Identifies Dysregulated Glycerophospholipid Metabolism in CRSwNP. **(A)** Volcano plot displaying differential metabolites in CRSwNP versus Normal. **(B)** Elevated levels of 1-stearoyl-2-arachidonoyl in CRSwNP versus Normal groups. **(C)** KEGG pathway enrichment of differential metabolites. **(D)** PCA score plot demonstrating separation between CRSwNP and Normal groups based on metabolomic profiles. **(E)** Metabolic heatmap analysis of CRSwNP vs. Normal. **(F)** ROC curve analysis of 1-stearoyl-2-arachidonoyl for distinguishing CRSwNP.

To further elucidate the metabolic alterations linked to CRSwNP, particularly the role of 1-stearoyl-2-arachidonoyl identified through Mendelian randomization, we performed metabolomic profiling comparing CRSwNP and healthy controls which were prospectively collected specifically for this study at Guangzhou Red Cross Hospital. Volcano plot analysis revealed significant upregulation of 1-stearoyl-2-arachidonoyl in CRSwNP (Figure 2A), confirmed by elevated levels in boxplot visualization (Figure 2B). KEGG pathway enrichment identified glycerophospholipid metabolism and cysteine-methionine metabolism as significantly dysregulated pathways (Figure 2C), consistent with 1-stearoyl-2-arachidonoyl’s role as a key glycerophospholipid. 1-stearoyl-2-arachidonoyl is a diacylglycerol species within the glycerophospholipid pathway, serving as a precursor for arachidonic acid release and subsequent eicosanoid production, which are known to modulate inflammatory responses in mucosal tissues. Principal component analysis (PCA) demonstrated clear separation between groups (Figure 2D), indicating distinct metabolic signatures. Hierarchical clustering of metabolites further corroborated disease-associated patterns (Figure 2E). Receiver operating characteristic (ROC) analysis revealed exceptional diagnostic performance for 1-stearoyl-2-arachidonoyl (AUC = 0.987, Figure 2F), supporting its biomarker potential. These results establish glycerophospholipid metabolic dysregulation as a hallmark of CRSwNP, highlighting 1-stearoyl-2-arachidonoyl’s central role.

### 3.3 Single-cell profiling and B Cell heterogeneity in CRSwNP

**FIGURE 3:**
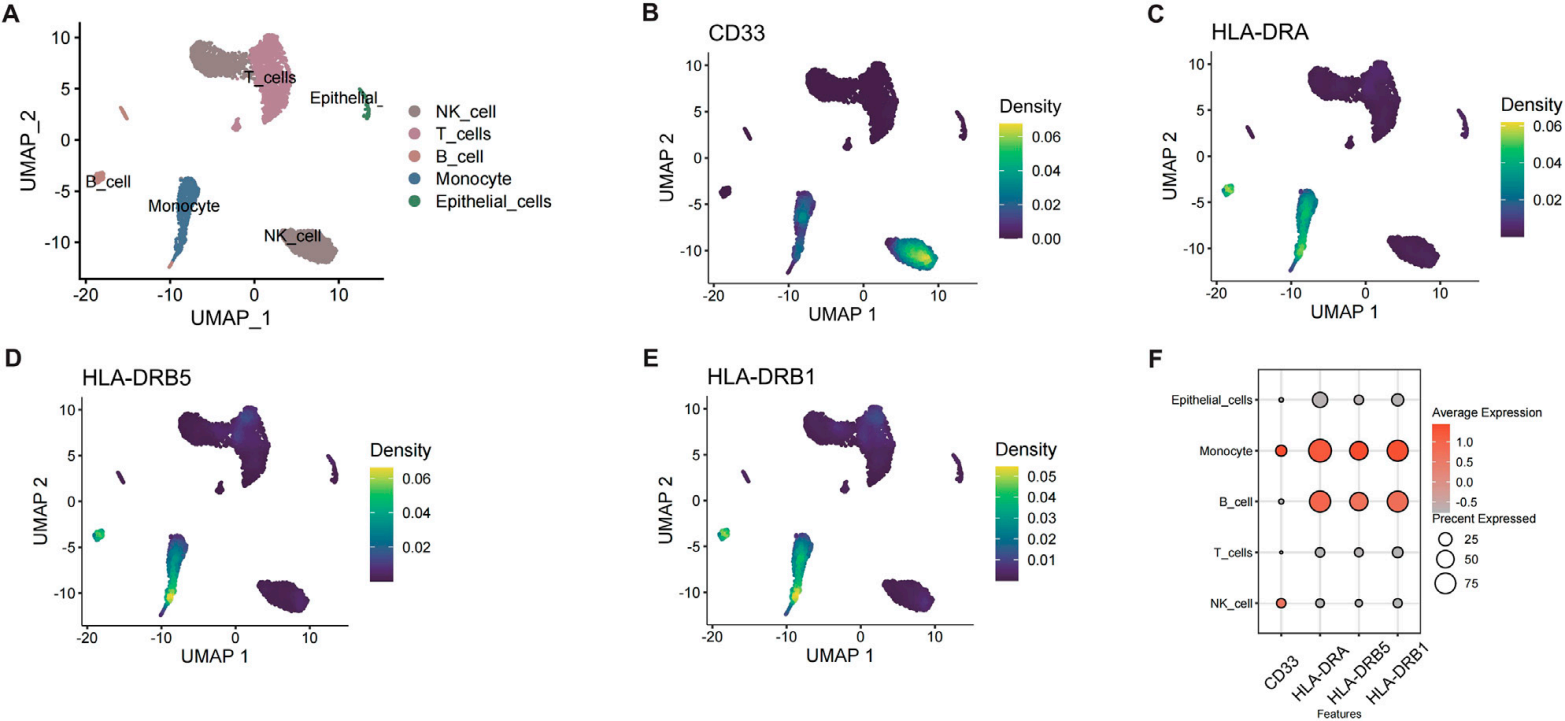
Single-cell RNA-seq analysis of B cells in CRSwNP. **(A)** UMAP plot of different cell subpopulations in CRSwNP. **(B)** Expression of CD33 in CRSwNP. **(C)** Expression of HLA-DRA in CRSwNP. **(D)** Expression of HLA-DRB5 in CRSwNP. **(E)** Expression of HLA-DRB1 in CRSwNP. **(F)** Expression of key genes in different cells.

Single-cell RNA sequencing (scRNA-seq) of CRSwNP samples (GSE196169) was performed to elucidate the roles of HLA-DR^+^CD33^−^ B cells and 1-stearoyl-2-arachidonoyl. After quality control and normalization, cell clustering identified five major populations: NK cells, T cells, B cells, monocytes, and epithelial cells (Figure 3A). Expression patterns of MR-implicated genes (CD33, HLA-DRA, HLA-DRB5, HLA-DRB1) were visualized across cell types (Figures 3B–E), confirming enrichment in the B cell compartment. Based on MR results for 1-stearoyl-2-arachidonoyl, we computed metabolic activity scores for glycerophospholipid pathway genes (LCAT, PLA2G4, PLA2G6, PLA2G16, PLB1, TGL4, DAGD, LCAT3) within B cells. Unsupervised clustering stratified B cells into two distinct subpopulations: PC_high_B_cell (elevated phospholipid metabolism) and PC_low_B_cell (Figure 3F).

### 3.4 Characterization of PC_high_B_cell-Associated pathways and interactions

**FIGURE 4:**
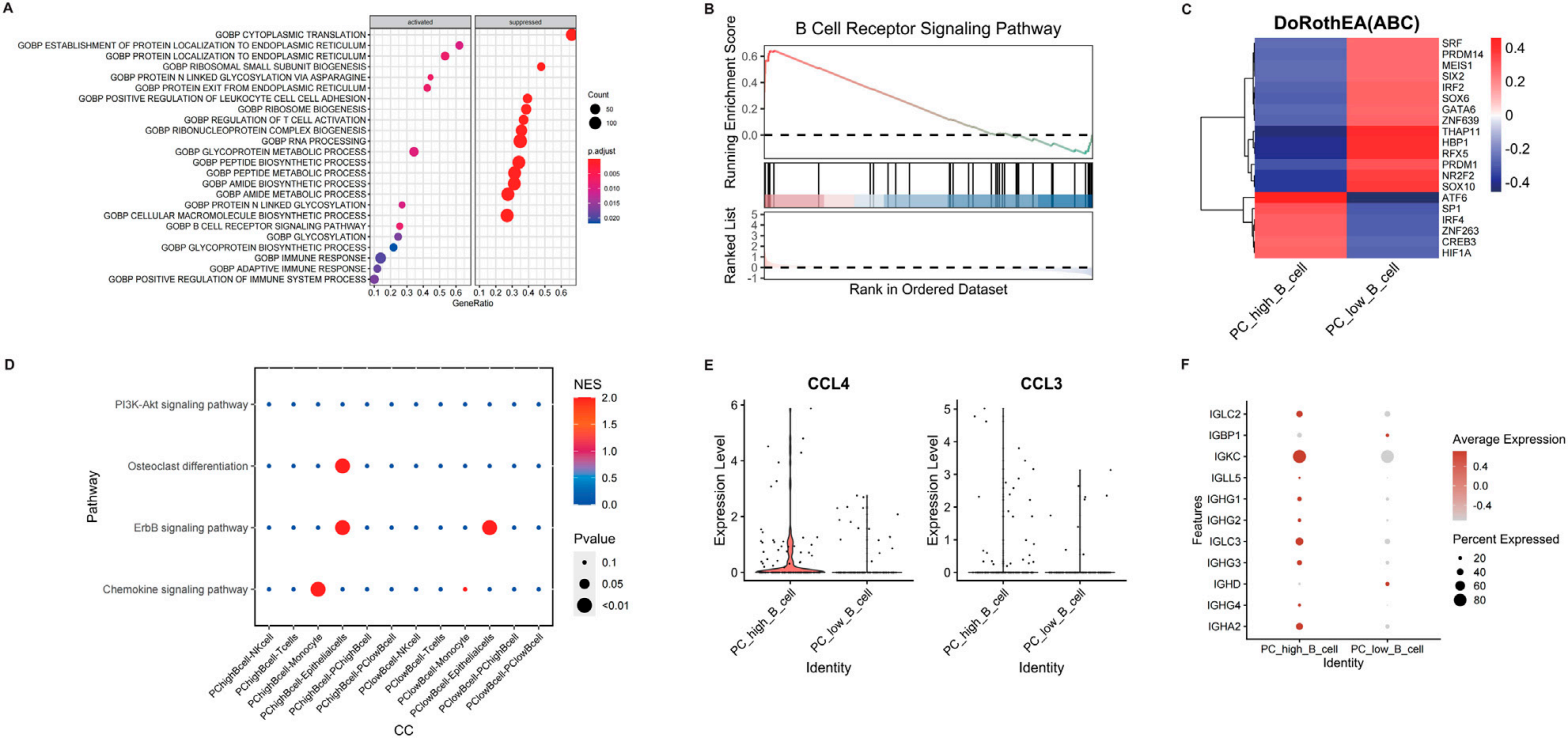
Differential analysis of different B cell subpopulations in CRSwNP. **(A)** GSEA enrichment analysis of differentially expressed genes in B cell subpopulations. **(B)** Analysis of B cell receptor pathway expression. **(C)** Transcription factor analysis of B cell subpopulations. **(D)** Cell-cell communication analysis of different cell subpopulations using CellCall. **(E)** Expression of CCL4 and CCL3 in different B cell subpopulations. **(F)** Expression of immunoglobulin-related genes in different B cell subpopulations.

Differential gene analysis of B cell subpopulations (PC_high_B_cell vs. PC_low_B_cell) revealed enrichment in immune response pathways via GSEA, including adaptive immunity and immune system activation (Figure 4A). Specifically, upregulated B-cell receptor signaling pathways suggested potential metabolite-receptor interactions modulating B cell function (Figure 4B). Transcription factor profiling identified elevated activity of ATF6, SP1, IRF4, ZNF263, CREB3, and HIF1A in PC_high_B_cells (Figure 4C). Intercellular communication analysis demonstrated preferential interactions between PC_high_B_cells and monocytes/epithelial cells, with signaling enriched in ErbB and chemokine pathways (Figure 4D). This was corroborated by significant upregulation of chemokines CCL3 and CCL4 alongside enhanced antibody production in PC_high_B_cells (Figures 4E,F), indicating metabolite-dependent B cell activation contributes to CRSwNP pathogenesis.

### 3.5 Bulk RNA-seq analysis of CRSwNP differential genes

While MR highlighted HLA-DR and CD33 as surface markers on B cells, scRNA-seq and bulk RNA analyses revealed downstream effectors such as CD27, DERL3, and TNFRSF17, which are functionally linked to B cell activation and survival in the context of elevated glycerophospholipid metabolism. Building on the single-cell findings, we analyzed bulk RNA-seq datasets (GSE36830 and GSE23552) to validate differentially expressed genes. After batch effect correction (Supplementary Figure S1A,B), differential expression analysis using limma identified significant alterations: NUCB2 was downregulated while CD27 and DERL3 were upregulated in CRSwNP (Supplementary Figure S1C–E). Correlation analysis revealed co-expression patterns among key genes (Supplementary Figure S2A–C), with pairwise comparisons confirming positive correlations between DERL3-CD27 and FKBP11-CD27 (Supplementary Figure S2D,E). These findings demonstrate coordinated upregulation of CD27 and DERL3 in CRSwNP, suggesting their potential role in disease pathogenesis. These DEGs align with MR-identified B cell subsets and metabolite dysregulation, supporting the hypothesis that 1-stearoyl-2-arachidonoyl drives B cell-mediated inflammation via genes involved in activation (CD27) and stress responses (DERL3).

### 3.6 Machine learning model for CRSwNP diagnosis

**FIGURE 5:**
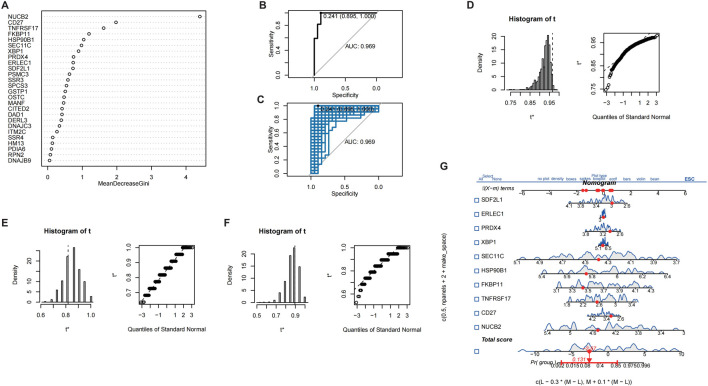
Screening of characteristic genes in CRSwNP. **(A)** Machine learning-based screening of characteristic genes in CRSwNP. **(B)** ROC curve of the results from multiple logistic regression. **(C)** Bootstrap validation combined with ROC. **(D)** Display of model bootstrap results. **(E)** Model sensitivity results. **(F)** Model specificity results. **(G)** Nomogram of different characteristic genes.

**FIGURE 6:**
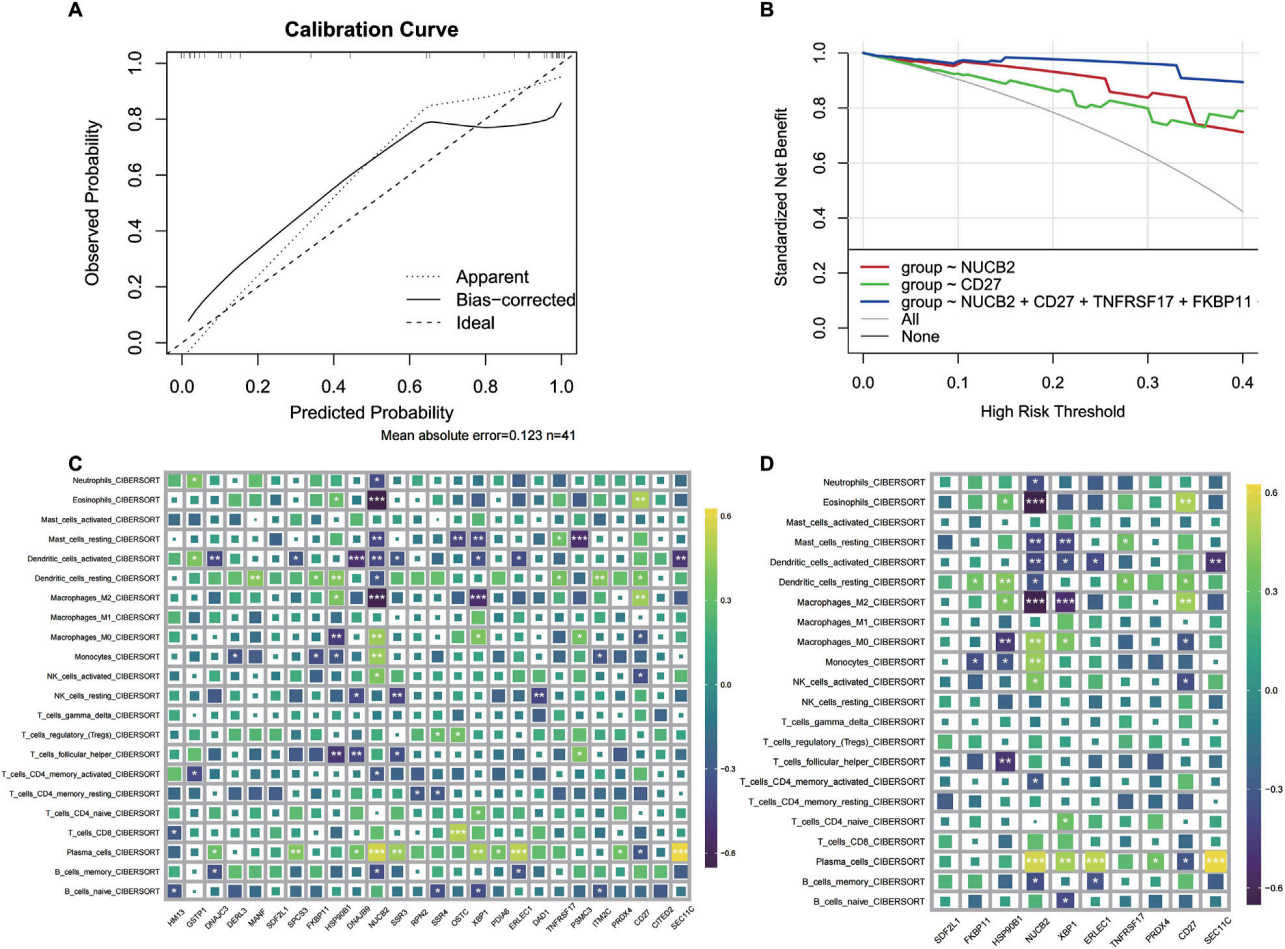
CRSwNP model prediction results and immune infiltration analysis. **(A)** Calibration curve of the model. **(B)** Decision curve of the regression model. **(C)** Infiltration analysis of differentially expressed genes. **(D)** Immune infiltration analysis of characteristic genes.

In order to further validate gene signatures, we developed a diagnostic model using random forest to identify key discriminative features. Feature importance ranking by Mean Decrease Gini identified 10 pivotal genes, including NUCB2 and CD27 (Figure 5A). Multivariable logistic regression modeling with these genes demonstrated high discriminative capacity (AUC = 0.969; Figures 5B,C), validated through bootstrap resampling with sensitivity/specificity quantification (Figures 5D–F). A nomogram visualizing predictor contributions (100 resamples, total score threshold = 0.131) and calibration curve (minimal deviation from ideal 45° line; Figure 6A) confirmed model robustness. Decision curve analysis comparing three gene-set models further established clinical utility (Figure 6B).

### 3.7 Immune microenvironment characterization via key gene signatures

**FIGURE 7:**
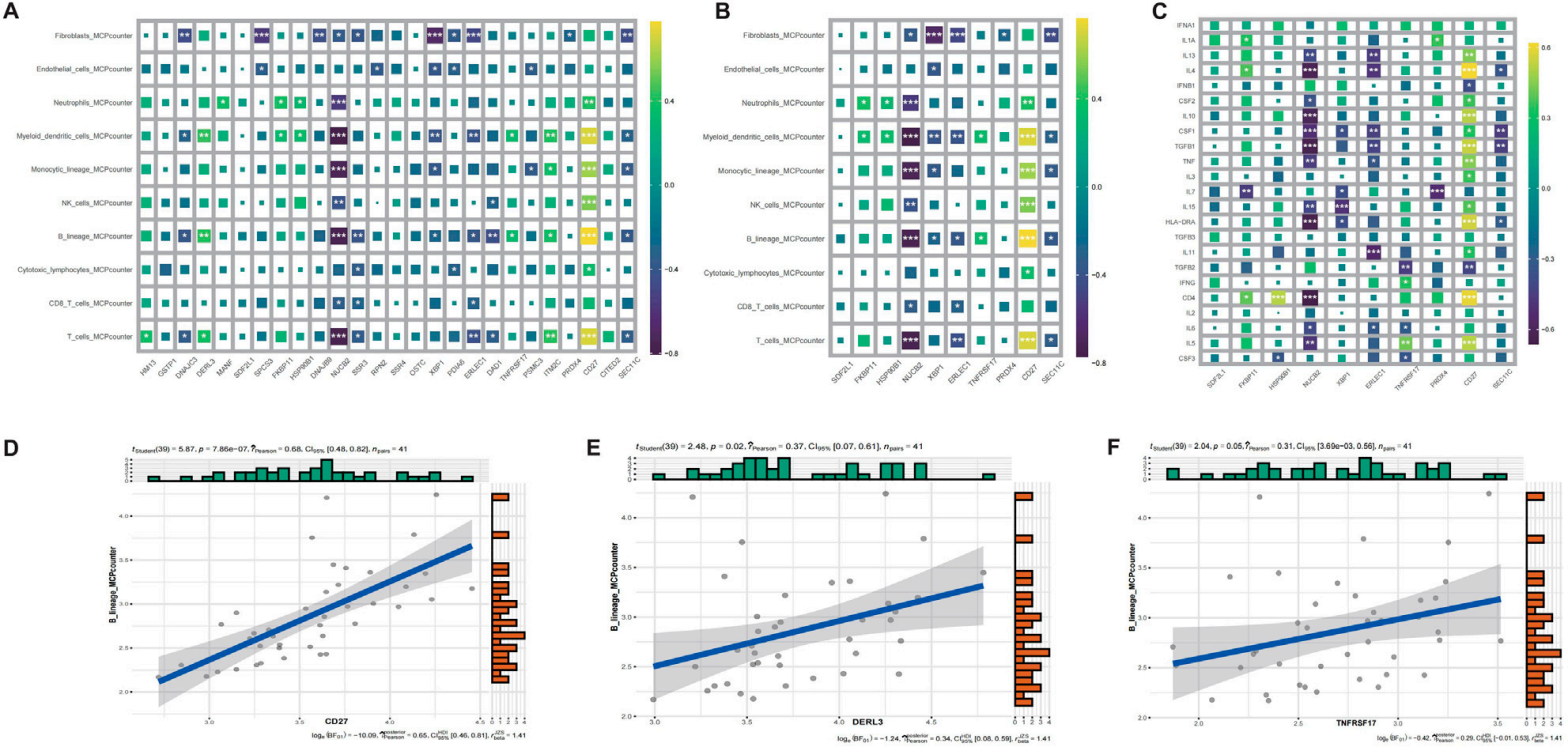
MCPcounter immune infiltration and correlation analysis of CRSwNP genes. **(A)** MCPcounter quantitative infiltration analysis of differentially expressed genes. **(B)** MCPcounter quantitative infiltration analysis of characteristic genes. **(C)** Inflammatory factor infiltration analysis of characteristic genes. **(D)** Correlation analysis between CD27 and B cell expression. **(E)** Correlation analysis between DERL3 and B cell expression. **(F)** Correlation analysis between TNFRSF17 and B cell expression.

To delineate the immunomodulatory role of identified key genes, we performed comprehensive immune infiltration analysis using CIBERSORT and ssGSEA (Figures 6C,D). Quantitative assessment via MCP-counter revealed distinct immune cell abundance patterns associated with CD27, DERL3, and TNFRSF17 expression (Figures 7A,B). Inflammatory factor profiling demonstrated significant correlations between CD27 and Th2 cytokines (IL13, IL4; Figure 7C), while B lineage-specific analysis showed positive associations of CD27, DERL3, and TNFRSF17 with B cell infiltration (Figures 7D,E). These findings suggest coordinated regulation of PC_high_B_cell functionality through metabolic-immunological crosstalk.

### 3.8 Molecular subtyping and Co-expression network analysis

**FIGURE 8:**
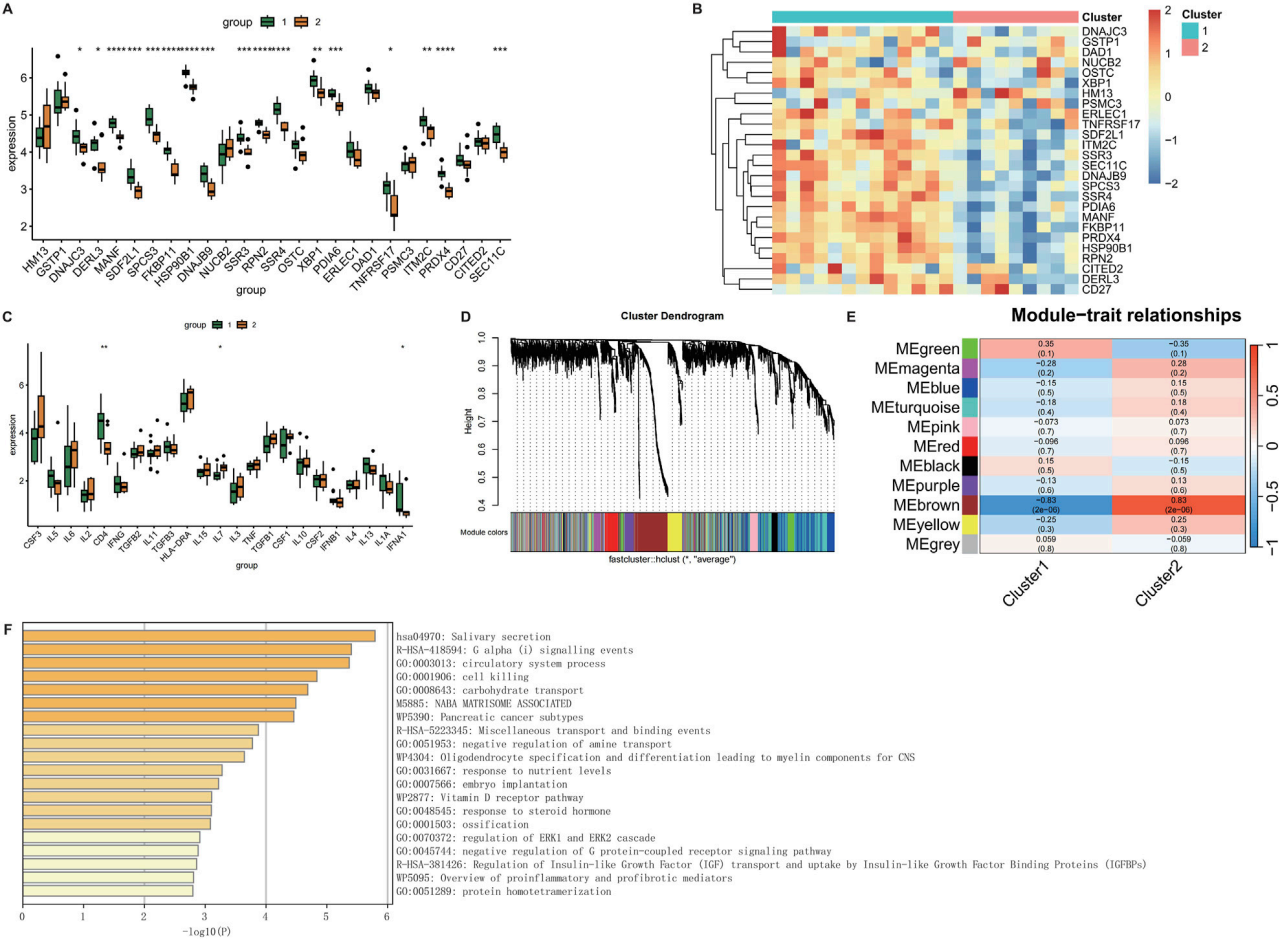
WGCNA analysis of CRSwNP. **(A)** Box plot of gene expression for clustering results. **(B)** Heatmap of gene expression for clustering results. **(C)** Box plot of clustering results for inflammatory factors. **(D)** WGCNA clustering dendrogram. **(E)** Heatmap of phenotype-gene correlations in WGCNA. **(F)** Metascape enrichment analysis of genes in the MEgreen module of WGCNA.

To define molecular subtypes associated with CRSwNP pathogenesis, consensus clustering identified two stable subgroups (k = 2) using k-means algorithm (100 iterations, kmax = 10). Cluster 1 exhibited elevated expression of key genes, while Cluster 2 showed reduced expression (Figures 8A,B). Inflammatory factor clustering further revealed differential abundance of CD4, IL7, and IFNA1 between subtypes (Figure 8C). Weighted gene co-expression network analysis (WGCNA) identified functionally cohesive modules, with soft-thresholding power set to 5 based on scale-free topology criteria (Figure 8D). The green module demonstrated highest immune relevance (Figure 8E), with Metascape enrichment confirming involvement in pro-inflammatory responses and cytotoxic regulation (Figure 8F; Supplementary Table S3).

## 4 Discussion

CRSwNP constitutes a persistent inflammatory disorder distinguished by mucosal hyperplasia and polyp formation within the sinonasal cavity, frequently characterized by chronicity, elevated recurrence rates, and heterogeneous responses to treatment (Nakayama and Haruna, 2023). These clinical complexities arise from sustained inflammation that precipitates pathological remodeling, thereby perpetuating symptom burden and rendering therapeutic management challenging. Through an integrated multi-omics approach—incorporating Mendelian randomization, single-cell transcriptomics, and bulk RNA sequencing—we have elucidated dysregulated immunometabolic pathways fundamental to the pathogenesis of CRSwNP. Specifically, our investigation delineates causal contributions of HLA-DR+CD33− B cell subsets and the glycerophospholipid metabolite 1-stearoyl-2-arachidonoyl, thereby unveiling novel mechanistic associations between metabolic reprogramming and immune dysregulation in this refractory condition.

The etiology of CRSwNP is multifaceted, involving factors such as infection, allergy, environmental pollution, and anatomical abnormalities. The role of immunity in its pathogenesis has increasingly come under scrutiny (Schleimer, 2017). SResearch by Shigeharu Fujieda et al. indicates that the activation of eosinophil-associated functions may contribute to the development of nasal polyps (Fujieda et al., 2019), while Jacob G. Eide et al. identified elevated levels of functionally active antiphospholipid antibodies in CRSwNP (Eide et al., 2022). Additionally, Kathryn E. Hulse et al. demonstrated that nasal polyp tissue creates an environment conducive to B cell survival and functionality, thereby facilitating disease progression (Hulse et al., 2013). To further elucidate the immunological mechanisms driving CRSwNP, we utilized Mendelian randomization and single-cell analysis to explore the causal links between immune cells and the condition. Our findings highlight the critical role of B cells in CRSwNP, confirming their contribution to disease advancement.

As key players in the adaptive immune response, B cells not only produce antibodies and cytokines that exacerbate CRSwNP pathogenesis but also participate in inflammation as antigen-presenting or regulatory cells (Tan et al., 2018). These cells secrete an array of cytokines, including IL-4, IL-5, IL-13, and chemokines, which amplify the inflammatory response and promote granulocyte activation and aggregation. This aligns with findings by Gwanghui Ryu, who noted that B-cell activators drive the progression of refractory CRSwNP through Th17-mediated immune responses and neutrophil recruitment (Ryu et al., 2019). Moreover, B cells serve as antigen-presenting cells, delivering antigens to T cells and triggering adaptive immune responses that result in further inflammatory cell infiltration. The chronic airway inflammation characteristic of CRSwNP fosters a unique milieu that supports B cell activation and antibody production (Feldman et al., 2017). Concurrently, activated mast cells in CRSwNP can stimulate B cells to produce IgE, intensifying the inflammatory cascade (Zhai et al., 2018). Another study revealed that the B cell-activating factor of the TNF family (BAFF) promotes IgA production and eosinophil activation, with increased presence of both naïve and effector B cell subtypes in CRSwNP (Miljkovic et al., 2018). Together, these findings underscore the indispensable role of B cells in both nasal mucosal health and the pathogenesis of CRSwNP.

Metabolic dysregulation plays a pivotal role in the pathogenesis of CRSwNP, as metabolites are known to modulate immune cell function and thereby influence inflammatory processes. Through Mendelian randomization analysis, we identified a causal relationship between the lipid metabolite 1-stearoyl-2-arachidonoyl and CRSwNP, with this metabolite exacerbating the condition. Composed of stearic acid and arachidonic acid, 1-stearoyl-2-arachidonoyl is integral to signaling pathways and inflammatory responses, acting as a key mediator that receives upstream signals and activates downstream inflammatory cascades (Xu et al., 2022). This finding aligns with research by Hao et al., who, through transcriptomic and metabolomic studies, demonstrated that 1-stearoyl-2-arachidonoyl coordinates the release of inflammatory cytokines (Hao et al., 2023). To further explore the effects of this metabolite, we conducted cell subpopulation annotation and differential gene expression analysis based on varying metabolic levels of 1-stearoyl-2-arachidonoyl. Our results revealed that B cell subpopulations exhibiting high metabolism of this lipid are closely associated with immune response pathways, including adaptive immune responses and positive regulation of the immune system, suggesting a pro-inflammatory role in CRSwNP. Additionally, differential gene analysis indicated significant upregulation of CD27, DERL3, and TNFRSF17 in these subpopulations, highlighting their involvement in the pro-inflammatory functions driven by 1-stearoyl-2-arachidonoyl metabolism.

CD27, a cell surface molecule belonging to the tumor necrosis factor receptor (TNFR) superfamily, plays a critical role in immune responses by promoting B cell survival and antibody production (Grimsholm, 2023). Upon antigenic stimulation, CD27^+^ B cells differentiate more efficiently into plasma cells and generate antibodies, a process facilitated by the interaction between CD27 and its ligand CD70, which provides co-stimulatory signals that enhance B cell activation and proliferation (Borst et al., 2005). Additionally, CD27 has been implicated in the differentiation of mouse B cells into memory B cells (Raman et al., 2003), and it directly drives the synthesis of IgG and IgM, both of which are essential in inflammatory processes. Notably, IgM+CD27+ B cells exhibit immunomodulatory functions and serve as a significant source of IL-10 (Sun et al., 2019), further underscoring the multifaceted role of CD27 in B cell-mediated immunity.

DERL3 (Derlin-3), a member of the Derlin family involved in protein folding and cellular stress responses, has been shown to exacerbate inflammation through the activation of NF-κB (Geng et al., 2020). Research by Li et al. also highlights DERL3’s close association with immune regulation, particularly in adaptive immune response and immune response regulation pathways (Li et al., 2020), which aligns with our findings. Similarly, TNFRSF17 (Tumor Necrosis Factor Receptor Superfamily, Member 17), also known as B-Cell Maturation Antigen (BCMA), is a TNFR superfamily member predominantly expressed on mature B cells and plasma cells. It regulates B cell survival, differentiation, and function, with BAFF mediating B cell activation through BCMA (Saltzman et al., 2013). Furthermore, TNFRSF17/BCMA is involved in antibody production and the formation of immune memory (Sabat et al., 2023), emphasizing its significance in sustaining immune responses. Together, the upregulation of CD27, DERL3, and TNFRSF17 in B cells within CRSwNP underscores their collective contribution to the pro-inflammatory milieu and immune dysregulation characteristic of this condition.

While our integrated multi-omics approach provides robust evidence for immunometabolic dysregulation in CRSwNP, several limitations should be acknowledged. Mendelian randomization offers evidence of potential causal associations but may be affected by pleiotropy or population stratification, despite FDR correction. The metabolomics analysis used a small sample size (12 controls and 19 patients), potentially limiting generalizability due to variability in collection and metabolite stability. Public datasets for scRNA-seq (GSE196169) and bulk RNA-seq (GSE36830, GSE23552) introduce risks of batch effects and heterogeneity from different platforms or populations. Inferences linking 1-stearoyl-2-arachidonoyl to B cell functions rely on correlations, which could be influenced by unmeasured confounders. No functional experiments (e.g., *in vitro* validations) were performed to confirm mechanisms. Future studies with larger, multi-ethnic cohorts and experimental validations are needed to enhance these findings.

## 5 Conclusion

Our study underscores the critical interplay between immune and metabolic processes in the pathogenesis of CRSwNP. Notably, the metabolite 1-stearoyl-2-arachidonoyl has been shown to bind to B-cell receptors, thereby enhancing inflammatory responses. Additionally, our findings highlight the significant roles of genes such as CD27, DERL3, and TNFRSF17 in shaping the immune microenvironment, elucidating specific pathological mechanisms underlying CRSwNP. A deeper comprehension of these molecular and cellular pathways offers a robust foundation for the development of personalized therapeutic strategies, with the potential to optimize clinical management and improve patient outcomes.

## Data Availability

The metabolomic data have been uploaded to MetaboLights (accession number: MTBLS13192), available at https://www.ebi.ac.uk/metabolights/editor/MTBLS13192/descriptors. Further inquiries can be directed to the corresponding author.

## Supplementary material

Supplementary Figure S1 Gene expression analysis of CRSwNP. **(A)** PCA before data integration. **(B)** PCA after data integration. **(C)** Heatmap of gene expression in CRSwNP group versus normal group. **(D)** Volcano plot of gene expression in CRSwNP group versus normal group. **(E)** Box plot of gene expression in CRSwNP group versus normal group.

Supplementary Figure S2 Correlation analysis of differentially expressed genes in CRSwNP. **(A)** Correlation analysis of differentially expressed genes across all groups. **(B)** Correlation analysis of differentially expressed genes in the CRSwNP group. **(C)** Correlation analysis of significantly differentially expressed genes. **(D)** Correlation analysis of DERL3 and CD27. **(E)** Correlation analysis of FKBP11 and CD27.

## Abbreviations

CRS, chronic rhinosinusitis; CRSwNP, Chronic rhinosinusitis with nasal polyps; ECRSwNP, eosinophilic Chronic rhinosinusitis with nasal polyps; non-ECRSwNP, non-eosinophilic chronic rhinosinusitis with nasal polyps; RVD1, resolvin D1; SPM, specialised pro-resolving lipid mediators; IVW, inverse variance weighting; AUC, Area Under Curve; Km, K-means; RFE, Recursive Feature Elimination; BAFF, B cell-activating factor of the TNF family; TNFR, tumor necrosis factor receptor.
