# Supplementary figures and images for: 1-stearoyl-2-arachidonoyl-driven B Cell metabolic dysregulation in chronic rhinosinusitis with nasal polyps: insights from Mendelian randomization and single-cell RNA sequencing
Source: Front Pharmacol. 2025 Oct 30;16:1719897. doi: 10.3389/fphar.2025.1719897 (PMC12611945; doi:10.3389/fphar.2025.1719897)

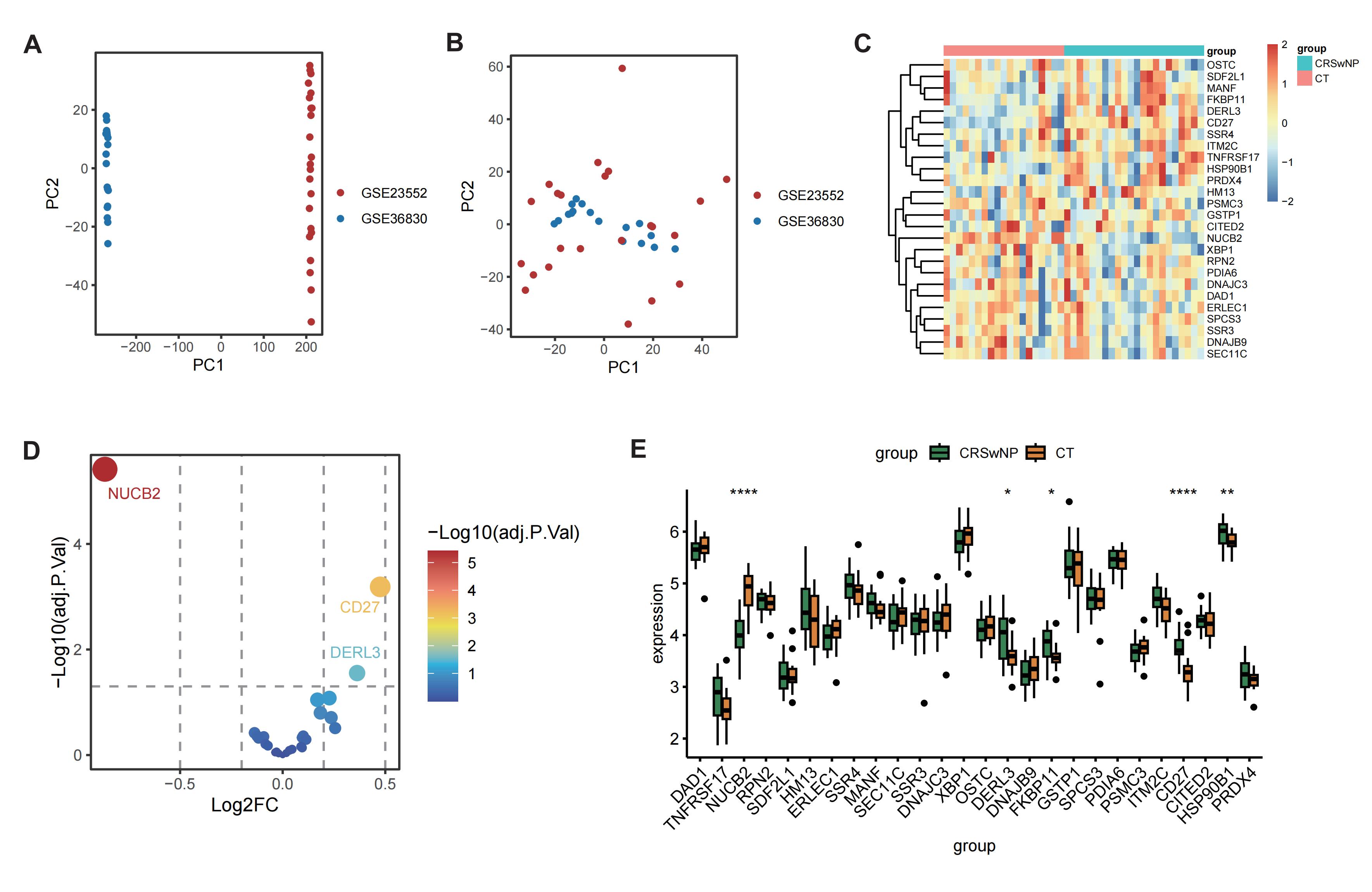

Supplement: Supplementary file 2 [file Image1.jpeg]

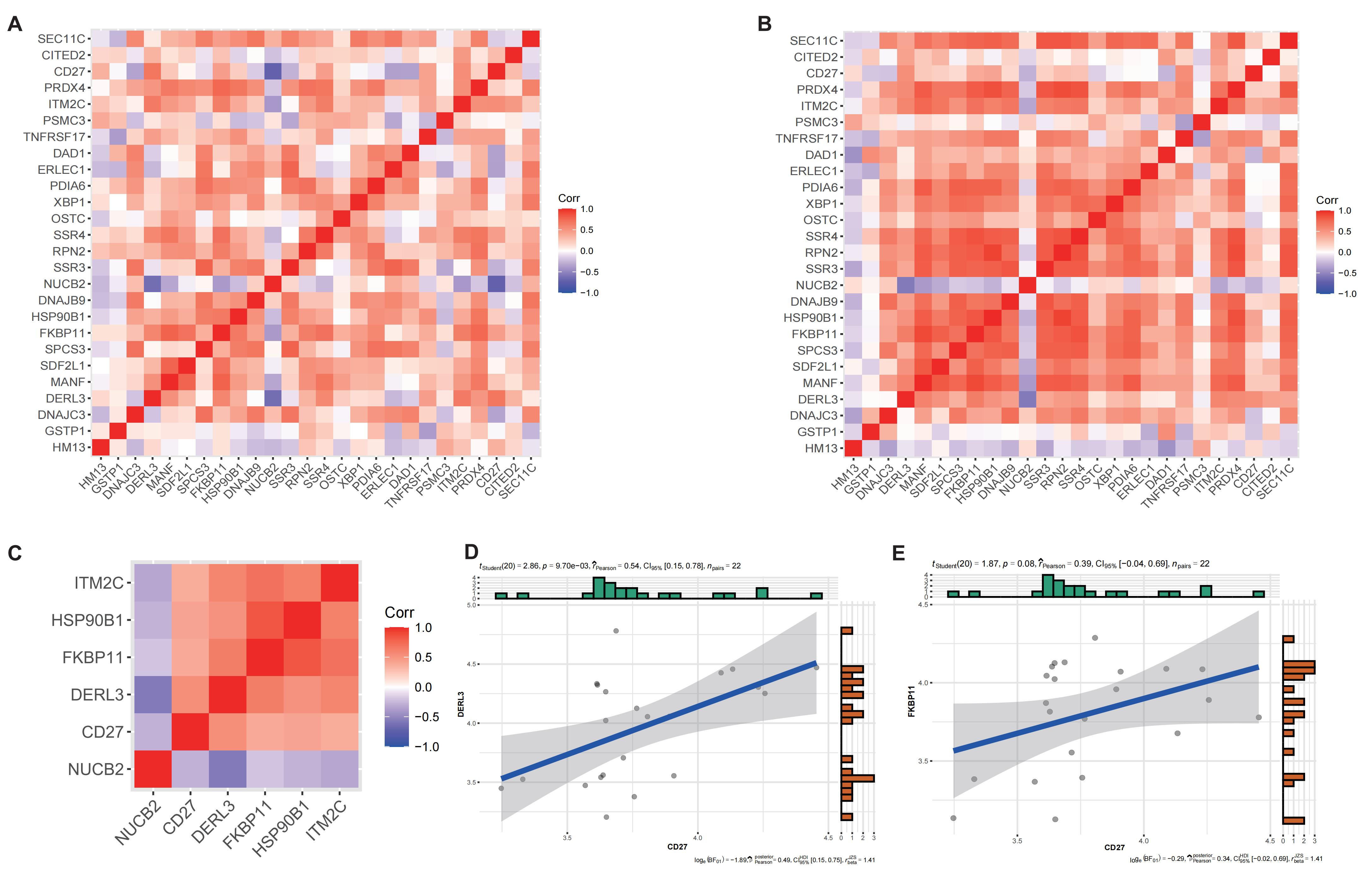

Supplement: Supplementary file 3 [file Image2.jpeg]
